# Electrochemical Sensors for Pesticide Residue Detection

**DOI:** 10.3390/molecules31101743

**Published:** 2026-05-20

**Authors:** Jiabin Sun, Xinjian Song, Yuan Zhang

**Affiliations:** 1 Hubei Key Laboratory of Biological Resources Protection and Utilization, School of Chemical and Environmental Engineering, Hubei Minzu University, Enshi 445000, China; 202330351@hbmzu.edu.cn; 2 College of Intelligent Systems Science and Engineering, Hubei Minzu University, Enshi 445000, China

**Keywords:** electrochemical sensors, pesticide residue, functional materials

## Abstract

Electrochemical sensors have emerged as promising tools for rapid pesticide screening in food and environmental samples because they combine simple instrumentation, fast response, portability, and compatibility with disposable electrodes. This review organizes recent progress through a cross-system framework linking pesticide class, interfacial electrochemical process, and material design. Carbon materials, metal–organic frameworks and their derivatives, metal nanoparticles, metal compounds, conducting polymers, MXene-based composites, and selected emerging materials are compared in terms of enrichment capability, charge-transfer regulation, catalytic amplification, recognition-layer integration, and suitability for real-sample analysis. Emphasis is placed on issues that are often under-discussed in performance-centered surveys, including matrix interference, electrode fouling, batch-to-batch reproducibility, storage stability, scalability, and cost-effectiveness. Representative examples show that the most useful advances arise not simply from lowering the limit of detection but from improving structure–function understanding and translating interfacial design into robust analytical performance. Future work should prioritize standardized fabrication and benchmarking protocols, in situ and operando identification of active sites and interface evolution, matrix-specific antifouling validation, multiresidue and metabolite analysis, and hybrid portable devices coupled with intelligent readout.

## 1. Introduction

Pesticides remain indispensable for safeguarding crop yields, improving the efficiency of pest, disease, and weed control, and supporting intensive agricultural production. However, their residual persistence, migration, and cumulative exposure in food, soil, and aquatic environments have kept pesticide detection at the intersection of food safety and environmental health research [1]. In monitoring pesticide residues, the central challenge is not merely whether they can be detected, but whether analysis can be performed rapidly, reliably, and at acceptable cost under complex matrices, low-dose conditions, and decentralized testing scenarios. Chromatography- and mass spectrometry-based techniques remain the core methods for confirmatory analysis and accurate quantification of pesticide residues [1,2]. Yet their dependence on elaborate sample pretreatment, sophisticated instrumentation, and skilled operation make them more suitable as laboratory benchmark tools than as practical solutions for high-frequency screening, on-site warning, or continuous monitoring [1,2]. Owing to their direct signal transduction, simplified instrumentation, and inherent suitability for miniaturization and portability, electrochemical sensing has emerged as an important methodological direction for rapid pesticide screening [3,4]. This transition indicates that the field is no longer defined solely by the pursuit of ever-lower detection limits, but is increasingly shaped by more practical constraints, including interfacial stability, compatibility with complex samples, device integrability, and translational potential [4,5].

The development of electrochemical detection for pesticides has progressed from direct responses at bare electrodes to the coordinated regulation of functional interfaces [3,6]. Early systems relied primarily on the direct redox behavior of target molecules at electrode surfaces, but limitations soon emerged owing to by-product adsorption, signal drift, and poor reproducibility. Research attention then shifted toward the coupling of electrode modification with recognition units, with analytical performance improved through the synergistic optimization of enrichment, recognition, catalysis, and charge transfer [3,5,6]. Within this evolution, materials innovation has provided the principal trajectory of methodological advancement. Carbon-based materials enhance electron transport and antifouling performance through their high electrical conductivity, large specific surface area, and modifiable surfaces [7]. Metal–organic frameworks (MOFs) and their derivatives strengthen molecular enrichment and catalytic performance by virtue of tunable pore structures, metal sites, and derivatization potential [8]. Metal nanoparticles, metal oxides, and other metal compounds further amplify electrochemical responses through heterogeneous interfaces, defect structures, and multivalent characteristics [9]. Conducting polymers and other nanostructured interfaces further expand interfacial tunability, while flexible substrates, microfluidic platforms, and portable terminals have pushed sensors beyond the stage of high-performance electrodes toward genuinely integrated devices [10,11,12,13]. Thus, the true logic of progress in this field does not lie in the continual replacement of one material family by another, but in how material characteristics reshape interfacial electrochemical behavior and ultimately determine analytical value.

Despite the rapid growth of related studies and review articles in recent years, several integration problems remain. Many recent reviews emphasize a single pesticide class, a single material family, or a single device format, which is useful for summarizing localized progress but less effective for revealing the shared principles that govern enrichment, recognition, charge transfer, catalysis, and real-sample performance across systems [7,12,14,15]. In addition, performance comparisons still rely too heavily on detection limits, linear ranges, and single-spike recoveries, while giving insufficient attention to matrix tolerance, long-term stability, batch-to-batch reproducibility, standardized fabrication, portability, and simultaneous multiresidue analysis [4,5,13]. As a result, the connection between material construction, interfacial electrochemical behavior, and practical analytical value is still not synthesized in a sufficiently unified way.

Against this background, the present review focuses on electrochemical sensing for pesticide residue detection from a cross-system and interface-engineering perspective. Rather than reiterating which individual material gives the lowest reported detection limit, we compare how different material classes regulate adsorption and enrichment, charge-transfer pathways, electrocatalytic amplification, recognition-layer organization, and sample compatibility. We first summarize pesticide classification, toxicological characteristics, and regulatory residue limits; we then outline the methodological boundaries of electrochemical sensing; and finally, we compare carbon-based materials, MOFs and their derivatives, metal nanoparticles, metal compounds, conducting polymers, MXene-based composites, and other emerging materials. This organization is intended to clarify the distinct contribution of the present review relative to recent material-specific or device-specific surveys by linking material characteristics, interfacial behavior, and translational analytical value within a single framework.

## 2. Pesticide Classification, Toxicity, and Maximum Permitted Limits

### 2.1. Classification

**Figure 1:**
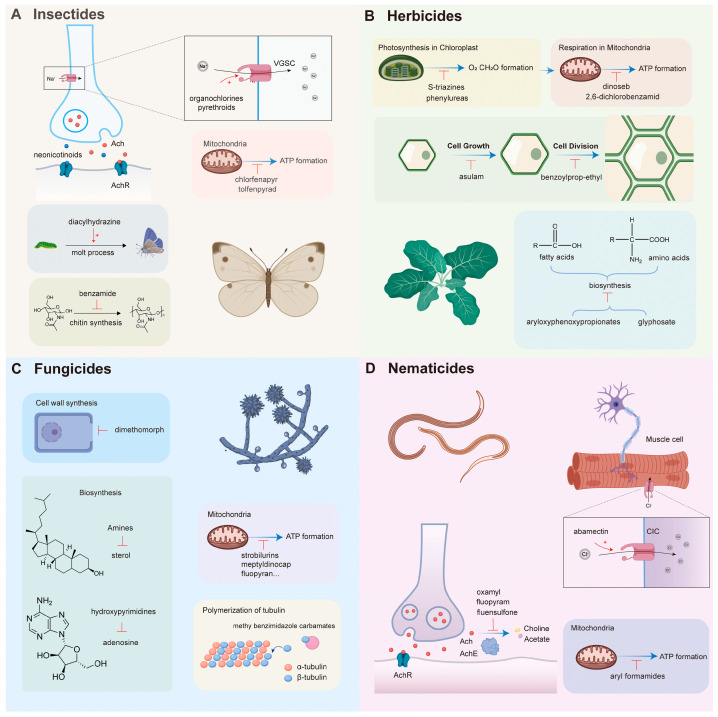
Representative mechanisms of pesticide action across the major classes discussed in this review. (**A**) Insectides. Ach: acetylcholine; AchR: acetylcholine receptors; VGSC: voltage-gated sodium channel. (**B**) Herbicides. (**C**) Fungicides. (**D**) Nematicides. AchE: acetylcholinesterase; CIC: chloride channels [17].

For studies of pesticide residues in food and environmental samples, three dimensions should be considered simultaneously: chemical structure, mode of action, and typical residue scenario [1,3,14,15,16]. As shown in Figure 1, pesticides can be classified as insecticides, herbicides, fungicides, and nematicides [17]. Representative classes, structural motifs, agricultural functions, and analytical implications are summarized in Table 1. Organophosphorus pesticides, carbamates, pyrethroids, neonicotinoids, phosphonate herbicides, and benzimidazole/triazole fungicides are among the most frequently discussed targets in current residue analysis [14,15,16]. This structure–mechanism framework is analytically important because it helps explain not only toxicological differences, but also differences in adsorption and enrichment behavior, electron-transfer characteristics, and signal-transduction pathways at electrochemical interfaces [3,5].

Organophosphorus pesticides and carbamates remain central to electrochemical pesticide sensing because their cholinesterase-inhibition pathways are well defined and therefore compatible with enzyme-inhibition biosensing [3,14]. Pyrethroids and neonicotinoids, by contrast, usually require stronger emphasis on selective recognition, interfacial enrichment, and peak deconvolution because their residue behavior and coexisting interference patterns differ substantially from classical cholinesterase inhibitors [1,15,16]. Herbicides and fungicides are even more heterogeneous: highly polar phosphonate herbicides such as glyphosate (Gly) are difficult to read directly because of weak electroactivity and poor hydrophobic enrichment [2,14], whereas bipyridylium herbicides and several benzimidazole fungicides are generally more amenable to direct or catalysis-assisted electrochemical transduction [1,3].

### 2.2. Toxicity and Exposure Characteristics

The differences among pesticide classes cannot be reduced to a simple ranking of “greater” or “lesser” toxicity. Rather, they arise from the combined effects of acute toxicity, chronic outcomes, environmental fate, bioaccumulation potential, and routes of dietary exposure. Organophosphorus pesticides and carbamates share a common analytical relevance because both are frequently discussed in relation to cholinesterase-associated toxicity and inhibition-based detection strategies [14,17]. Carbamate inhibition is generally more reversible, whereas some organophosphorus compounds, together with their oxon metabolites, are associated with stronger acute toxic concern and longer-standing regulatory scrutiny [17]. Pyrethroids, by contrast, are more often discussed analytically in terms of hydrophobicity, matrix partitioning, and residue-distribution behavior than in terms of cholinesterase-associated toxicity [1]. Neonicotinoids further illustrate that high target selectivity does not necessarily imply low risk. They are frequently detected in soils, water bodies, fruits, vegetables, and bee products, and may exert acute, sublethal, and chronic effects on pollinators, birds, aquatic organisms, and mammals, involving neurological, immunological, developmental, and reproductive endpoints [18]. For highly polar organophosphonate pesticides such as Gly, risk considerations relate not only to their widespread yet generally low-level occurrence in food and the environment, but also to the analytical reality that they are difficult to determine directly in diverse sample matrices [2,14]. For fungicides such as triazoles, by contrast, regulatory concern arises more from long-term low-dose exposure and the heterogeneity of toxicological evidence [19].

More fundamentally, persistence and mobility amplify the practical significance of toxicological differences in shaping analytical strategies. Although many organochlorine pesticides have already been banned or severely restricted in numerous regions, their strong environmental persistence and marked bioaccumulation mean that they remain important targets in the assessment of historical residues and background contamination [17]. By comparison, organophosphorus pesticides generally have shorter environmental half-lives yet continue to attract sustained attention in rapid food screening because of their widespread use, clearly defined biological targets, and pronounced acute toxic concern [17]. Pyrethroids and neonicotinoids reflect a different pattern, namely that replacement does not eliminate the need for reassessment. Neonicotinoids have continued to provoke scrutiny regarding non-target risks and environmental fate despite their extensive use and high target selectivity [18]. Static monitoring based solely on parent compounds is therefore no longer sufficient to capture real exposure scenarios in a complete manner. Instead, residue analysis with greater practical relevance increasingly requires attention to multiple coexisting residues, complex sample matrices, and, where necessary, the inclusion of metabolites and transformation products [17,18,19].

### 2.3. Permitted Limits

Codex defines the maximum residue limit (MRL) as the highest level of pesticide residue that is legally permitted in food or feed under conditions of good agricultural practice [20]. WHO guidance documents on the risk assessment of chemicals in food further clarify the roles of health-based guidance values, including the acceptable daily intake and acute reference dose, in regulatory evaluation [21]. Within the European Union, harmonized MRL management is implemented under Regulation (EC) No 396/2005, whereas the United States Environmental Protection Agency applies a tolerance system that is conceptually similar to the MRL framework but differs in legal pathway, commodity classification, and enforcement logic [22,23]. Accordingly, there is no single globally uniform standard for “maximum permitted limits”; the same pesticide may correspond to different regulatory thresholds depending on the commodity, jurisdiction, and risk-assessment framework considered [20,21,22,23].

From a methodological perspective, residue-limit systems directly shape the performance targets of pesticide analysis. The 2023 European Union report on pesticide residues in food, published by EFSA in 2025, shows that the coexistence of multiple residues has become a common reality [24]. This indicates that regulatory demand has already moved beyond the single-analyte question and now places increasing emphasis on quantitative reliability, selectivity, and the capacity for multicomponent interpretation in complex samples [24]. Within the EU framework, a default MRL of 0.01 mg/kg is generally applied when no specific limit has been established [22]. In the Codex system, limits for different pesticide-commodity combinations vary substantially depending on the analyte and commodity and may extend from low default-like levels to markedly higher values in specific pairings, especially for certain highly polar herbicides and major crops [20]. Differences spanning wide regulatory ranges make clear that subsequent analytical methods cannot regard an ultralow detection limit as their sole advantage. Rather, they must be evaluated in relation to the pesticide class of interest, the actual commodity matrix, and the applicable regulatory framework, with integrated consideration of sensitivity, quantitative accuracy, dynamic range, and the capacity for parallel multiresidue analysis. It is precisely in this context that electrochemical sensors remain worthy of continued development. Their value lies not merely in rapid response, simplified instrumentation, or lower cost, but in their potential to be coupled with functional materials, miniaturized devices, and portable terminals, thereby providing an effective complement to standard methods in complex samples and front-end screening scenarios [4,12,13].

## 3. Electrochemical Sensing

### 3.1. Introduction to Electrochemical Sensors

In pesticide residue analysis, electrochemical sensors are most useful as front-end screening tools rather than as replacements for chromatography- or mass-spectrometry-based confirmatory methods [1,3,13]. Their practical value lies in directly converting redox processes, electrocatalysis, enzyme inhibition, specific adsorption, or interfacial impedance changes into measurable electrical signals under relatively simple operating conditions [3,5,25]. Compact instrumentation, fast response, easy miniaturization, and compatibility with disposable or portable electrodes make electrochemical platforms attractive for food, environmental, and supply-chain monitoring, but their real usefulness still depends on selectivity in complex matrices, stability, reproducibility, and consistency with benchmark analytical methods [4,12,13].

### 3.2. Classification

**Figure 2:**
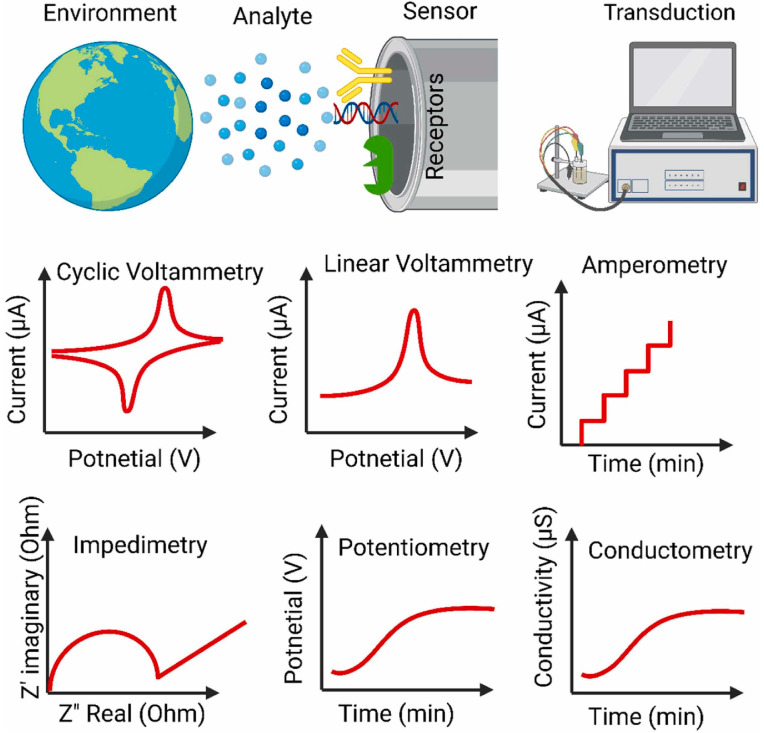
Schematic diagram of electrochemical signal transduction used in electrochemical sensors: voltammetry, amperometry, impedimetry, potentiometry, and conductometry [25].

From the perspective of signal readout, electrochemical sensors for pesticide detection can be grouped into voltammetric/amperometric, potentiometric, and impedimetric systems, as shown in Figure 2 [3,25,26]. Voltammetric and amperometric platforms dominate most reported pesticide studies because they are well suited to intrinsically electroactive analytes and to interfaces that amplify Faradaic signals through electrocatalysis [3,14,26,27,28]. Potentiometric approaches are more limited but remain useful for selected ionizable targets or inhibition-based systems [25]. Impedimetric sensors are especially valuable for label-free monitoring of binding events on antibody-, aptamer-, and molecularly imprinted interfaces [3,26].

According to the recognition mechanism, electrochemical pesticide sensors can be broadly classified as enzyme-based, affinity-based, or non-enzymatic electrocatalytic systems [3,25]. Enzyme-based sensors offer well-defined signal pathways and high sensitivity, but their long-term deployment is restricted by conformational fragility and storage requirements [3,14]. Affinity-based strategies, including antibodies, aptamers, and molecularly imprinted polymers, provide stronger molecular selectivity but still face challenges such as heterogeneous recognition sites, template leakage, and nonspecific adsorption [3,26]. Non-enzymatic platforms instead rely on interfacial design to promote enrichment, direct redox conversion, or catalytic signal amplification [5,27,28].

### 3.3. Challenges

In pesticide residue analysis, performance evaluation should not be restricted to the conventional pair of detection limits and linear range. These two metrics remain necessary because they define whether a sensor can reach regulatory thresholds and accommodate different contamination levels, but they are insufficient on their own. Selectivity in complex matrices, resistance to fouling and passivation, repeatability, batch-to-batch reproducibility, storage stability, response time, and validation in real samples are equally important when judging whether a platform has practical analytical value [5,13,25].

At present, the key bottlenecks in this field are concentrated at four levels. First, selectivity in complex matrices remains the most pervasive challenge. This is particularly evident in systems containing structurally similar pesticides, multiple coexisting residues, or samples with high organic loads, where a single interfacial modification is often insufficient to ensure both strong signal response and adequate specificity [5,13]. Second, contamination and passivation of electrode surfaces have not yet been fundamentally resolved. Many organic pesticides, or their oxidation by-products, tend to accumulate at the interface, leading in turn to peak distortion and declining repeatability [5,6]. Third, the excellent performance frequently reported in the literature is often established in ideal buffer systems, with single analytes and only limited recovery experiments in real samples; by contrast, convincing evidence remains insufficient with respect to large-sample validation in complex matrices, cross-batch reproducibility, and inter-platform consistency [4,5,12,13]. Fourth, although the simultaneous detection of multiple pesticides is an explicit requirement in real applications, marked differences among pesticides in electrochemical activity, response potential, enrichment behavior, and recognition mechanism continue to make stable, interpretable, and standardized multicomponent analysis difficult to achieve [5,12,13].

## 4. Applications of Active Materials in Electrochemical Sensing for Pesticide Residue Detection

The selection and design of electrode materials are central determinants of electrochemical sensor performance. In recent years, the development of functional nanomaterials has opened new avenues for constructing highly sensitive and highly selective sensing interfaces for pesticide residue detection. According to their chemical composition and structural characteristics, the electroactive materials used in this field can be broadly categorized into carbon-based materials, MOFs and their derivatives, metal nanoparticles, metal compound-based materials, conducting polymers, and MXene-based composites.

### 4.1. Carbon-Based Materials

Carbon-based materials are not merely generic conductivity enhancers; they act as interfacial platforms that regulate electronic structure, molecular enrichment, recognition-layer organization, and signal-transduction efficiency [7,16]. Their usefulness arises from conjugated conductive frameworks, large accessible surface areas, and surface defects or functional groups that enable catalytic tuning, immobilization, and composite construction [7]. Although different carbon allotropes display distinct morphologies, their analytical value ultimately depends on how their structures shape, charge transport, adsorption behavior, and interfacial accessibility.

**Figure 3:**
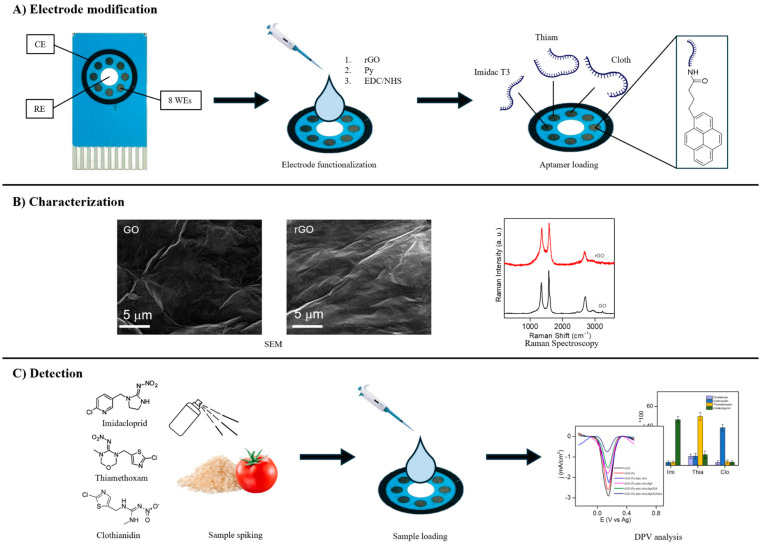
Reduced graphene oxide-based electrochemical aptasensor for the multiplexed detection of imidacloprid, thiamethoxam, and clothianidin in food samples [29].

Taking graphene and its derivatives as an example, the continuous sp^2^ carbon network is advantageous for constructing low-impedance signal pathways, yet restacking of nanosheets can diminish the accessible surface area and impair wettability. In practical systems, oxygen-containing groups, porous architectures, or heteroatom sites are therefore often introduced to balance electrical conductivity with interfacial tunability [29,30,31]. Almenhali and co-workers used a reduced graphene oxide platform to support multiplex aptamer recognition and achieved simultaneous detection of imidacloprid, thiamethoxam, and clothianidin in food samples, showing that the value of graphene derivatives lies not merely in amplifying peak currents, but more fundamentally in providing a scalable interface for ordered multitarget recognition-layer assembly and efficient interfacial charge transfer, as shown in Figure 3 [29].

The effectiveness of carbon nanotube-based systems likewise does not arise simply from “high conductivity” but from their ability to form continuous one-dimensional interconnected networks that maintain stable electronic percolation and mechanical integrity within polymers, ionic liquids, or metallic nanophases, thereby making them particularly suitable for flow analysis, flexible electrodes, and printable devices [32,33,34]. On this basis, the incorporation of electroactive groups such as thiophene and ferrocene further transforms carbon nanotubes from passive wire-like scaffolds into multifunctional interfaces capable of mediating charge transfer, supporting recognition layers, and amplifying signals [33].

Porous and biomass-derived carbons are distinguished more by the synergy among hierarchical pore architectures, defect structures, and surface chemistry. Micropores provide high densities of adsorption sites, mesopores and macropores shorten diffusion pathways and mitigate matrix blockage, while defects and heteroatom sites regulate the adsorption strength of target molecules and the rate of charge transfer [35,36,37,38]. These features make such materials particularly well suited to serving simultaneously as enrichment platforms, support for recognition, and antifouling buffer layers. Carbon and graphene quantum dots are less capable of independently establishing highly efficient continuous conductive networks, but their abundant carboxyl, hydroxyl, and amino groups, excellent aqueous dispersibility, and short-range electron-shuttling ability confer distinct advantages in interfacial stabilization, enzyme or aptamer immobilization, and the uniform construction of composite layers for organophosphorus pesticide sensing [39,40,41].

For nitroaromatic pesticides or organophosphorus compounds with relatively well-defined electroactivity, carbon-based interfaces can enable direct voltammetric detection through defect-induced electron transfer, π-mediated surface enrichment, and coupled catalysis with metals or metal oxides [30,31,32,33,34]. In systems that depend on recognition elements, carbon materials are more suitably regarded as efficient supporting platforms for aptamers, enzymes, or molecularly imprinted layers, with porous carbons preserving the accessibility of recognition layers, graphene and carbon nanotubes providing rapid electron-extraction pathways, and carbon dots improving hydrophilicity and immobilization uniformity [29,35,39,40,41]. Available studies indicate that carbon-based platforms are now capable of supporting a wide range of sensing strategies, including direct electrocatalytic detection, acetylcholinesterase (AchE) inhibition-based sensing, aptamer-based recognition, and multicomponent composite recognition. At the same time, their applicability has clear boundaries. For highly polar, weakly electroactive, or structurally similar pesticides, the carbon framework alone is often insufficient to address both selectivity and transduction efficiency, and effective sensing still requires coupling with phosphorus-affinitive sites, catalytic centers, or highly specific recognition layers [2,16,26].

Although carbon-based materials remain among the most important material systems in electrochemical sensing for pesticide residues, their practical limitations have become increasingly clear. First, restacking of graphene sheets, aggregation of carbon nanotubes, batch-to-batch variation in biomass-derived porous carbons, and heterogeneity of surface ligands on carbon dots can all lead to pronounced fluctuations in exposed active sites and electrochemical responses across different preparation batches [7,38,39,40,41]. Second, current discussions of defects, heteroatom doping, and pore structure still largely remain at the level of empirical generalization and often fail to disentangle the independent contributions of edge sites, vacancies, doping configurations, and composite catalytic phases. As a result, a clear disconnect often persists between reported performance enhancement and the underlying mechanism of action [5,7,36]. Third, carbon interfaces with high specific surface areas are commonly accompanied by stronger nonspecific adsorption. Proteins, pigments, polysaccharides, and humic substances in fruit and vegetable juices, environmental waters, and real agricultural extracts can readily induce fouling, signal drift, and false responses, making it difficult for many highly sensitive systems to maintain long-term stability in real samples [4,5,35,37,38]. In addition, many high-performance platforms rely excessively on multicomponent hybridization, such that the intrinsic contribution of the carbon phase is obscured by metals, MOF-derived materials, or complex interfacial amplification strategies, which in turn hampers the establishment of transferable design principles [5,7,16].

### 4.2. MOFs and Their Derivatives

The value of MOFs and their derivatives extends far beyond high specific surface area. Their metal nodes, organic linkers, and pore microenvironments can be engineered together so that target enrichment, recognition-element immobilization, interfacial catalysis, and charge transport are coupled within a single framework [8,42]. Compared with conventional inorganic porous materials, the most important feature of MOFs is structural programmability: node composition influences Lewis acidity/basicity and redox activity, linker structure influences pore polarity and host-guest interactions, and framework topology regulates diffusion pathways and interfacial accessibility [8,42].

Representative pristine MOF families such as UiO (University of Oslo)-type, MIL (Materials of Institut Lavoisier)-type, PCN (Porous Coordination Network)-type, and Cu-BTC (copper benzene-1,3,5-tricarboxylate) typically exhibit high porosity and abundant open metal sites, making them well suited for adsorbing phosphorus-containing, nitrogen-containing, or aromatic heterocyclic pesticide molecules [8,42]. However, what ultimately determines sensing performance is not surface area alone, but the interfacial environment collectively shaped by metal-site identity, linker polarity, pore-size distribution, and surface wettability. Cao and co-workers constructed a Gly sensor based on hierarchically porous Cu-BTC, showing that the key role of the pristine MOF was not simply to enlarge active areas, but to shorten diffusion pathways through hierarchical channels while enhancing interfacial enrichment efficiency through interactions between Cu sites and phosphonate groups [43].

**Figure 4:**
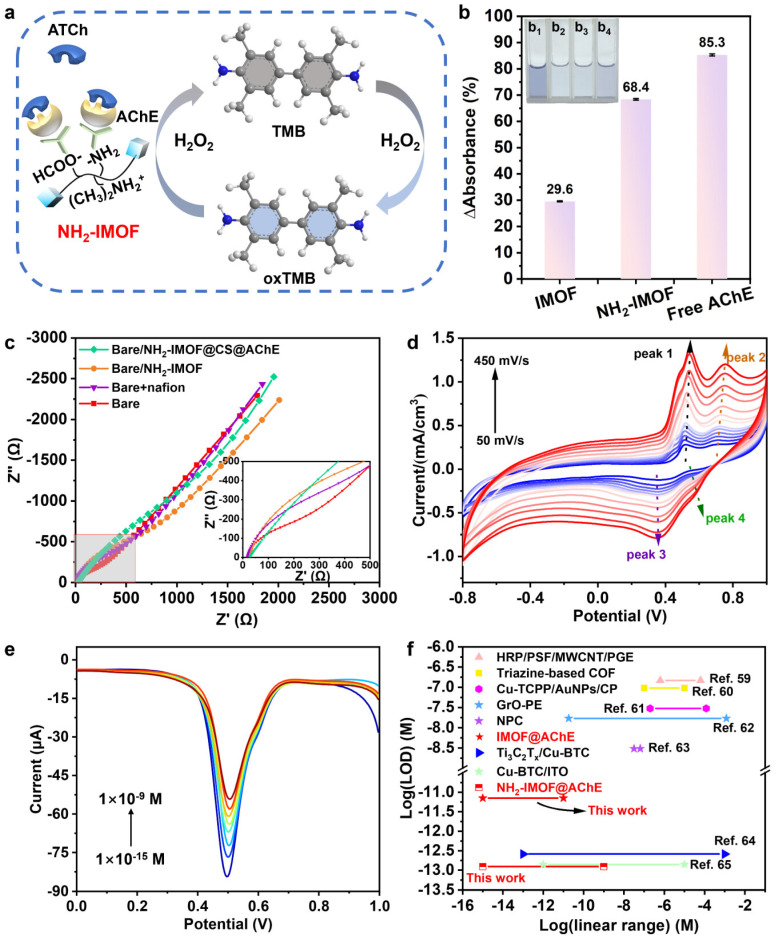
(**a**) Scheme of the change in enzyme activity after immobilization by using tetramethylbenzidine (TMB); (**b**) The degree of oxTMB discoloration in comparison after free-AChE, amino-functionalized ionic MOF (NH_2_-IMOF)@CS@AChE, and IMOF@CS@AChE addition, respectively. (Inset: After fading pictures of: b1 blank; b2 free-AChE; b3 NH_2_-IMOF@CS@AChE; b4 IMOF@CS@AChE); (**c**) EIS Nyquist plots of electrodes, inset shows expansion in the high-frequency range; (**d**) Influence of scan rate on peak current when using NH_2_-IMOF@CS@AChE as the sensor; (**e**) differential pulse voltammetry studies of NH_2_-IMOF@CS@AChE in PBS (0.1 M, pH 7.5) containing 2.5 mM ATCh with different concentrations of glyphosate (Gly); (**f**) Comparison of the NH_2_-IMOF@CS@AChE sensor performance for Gly detection with other literature [44].

Because pristine MOFs are limited in charge transport, conductive or ionic MOFs, multimetal MOFs, and MOF-based composites have become major directions of continued development [8,42]. In the context of electrochemical sensing for pesticides, improved conductivity does not refer only to faster electron mobility, but also to better local ion transport, surface charge compensation, and steady-state control of the recognition layer. As shown in Figure 4, the amino-functionalized ionic MOF (NH_2_-IMOF) reported by Wu and co-workers demonstrates that a MOF can simultaneously regulate the stability of AChE immobilization, substrate diffusion, and interfacial charge transfer, and can further be integrated with a portable readout module for on-site detection [44]. Multimetal or mixed-valence MOFs, in turn, reconstruct local electronic states and target-binding modes through node-level synergy.

From a translational standpoint, the most practically viable strategies currently remain MOF-based composite systems and MOF-derived materials. Duan and co-workers used curcumin-enhanced UiO-66 to construct a sensor for parathion-methyl, demonstrating that the incorporation of a natural small molecule does not merely serve as an auxiliary amplification step, but instead reconstructs the interfacial electron-transfer barrier through coupling with the enrichment interface provided by the MOF [45]. The MIP-MOF-808/acetylene black system reported by Zhang and co-workers shows that the selectivity of the imprinted layer does not depend exclusively on the polymer shell, because the pores of the MOF and the Zr sites also participate directly in recognition and enrichment [46]. Similarly, the AChE immobilization platform based on MnMOF developed by Tan and co-workers highlights the importance of charged channels and the interfacial microenvironment in maintaining the conformational stability of the enzyme [47]. By contrast, MOF-derived materials place greater emphasis on enhanced conductivity, exposure of active sites, and the reconstruction of hierarchical porous architectures. The MOF-derived porous carbon/graphene composite reported by Guo and co-workers, the MOF-derived CuCo_2_O_4_ heterointerface described by Maji and co-workers, and the hollow N-doped Cu-based MOF derivative developed by Zhao and co-workers all indicate that the most important contribution of derivatization lies in extending simple pore-based enrichment into a synergistic framework of enrichment, electron transport, and electrocatalysis [48,49,50]. Studies by Sun and Yang suggest that this route has already expanded beyond conventional organophosphorus pesticide detection to more complex analytical targets, including fungicides and insect growth regulators [51,52].

The effectiveness of MOFs and their derivatives in electrochemical sensing for pesticide residues arises from synergy across four levels: target enrichment driven by pore architecture and surface chemistry; interfacial catalysis provided by open metal sites, mixed-valence nodes, or derived active phases; improved charge transport enabled by conductive frameworks or ionic microenvironments; and the ability to immobilize recognition elements through modifiable surfaces and ordered pore structures [8,42,44]. At the same time, their practical boundaries are equally evident. First, the intrinsic conductivity and water stability of pristine MOFs remain widespread bottlenecks. In real fruit and vegetable extracts and aqueous buffer systems, framework hydrolysis, film detachment, and signal drift are still difficult to eliminate completely [8,42]. Second, mechanistic attribution remains insufficient with respect to which factors truly dominate sensor response. Open metal sites, defects, pore size, external conductive phases, and recognition-layer thickness often vary simultaneously, such that structure-performance relationships remain largely at the level of empirical correlation [5,8]. Third, evidence for antifouling performance and resistance to matrix interference in complex samples remains inadequate. Although many studies report satisfactory recovery values, systematic discussion of the long-term fouling effects of pigments, sugars, organic acids, and colloidal particulates is still limited. Finally, whether in pristine MOFs or derived materials, batch-to-batch consistency, scalable synthetic windows, and standardized characterization frameworks remain insufficiently mature [5,8,42].

### 4.3. Metal Nanoparticles

Metal nanoparticles contribute far more than a generic increase in electrode conductivity. Owing to nanoscale surface atoms, highly curved interfaces, and tunable electronic structures, they can reconfigure analyte adsorption, activation, charge transfer, and signal generation, thereby serving as electrocatalytic active centers, signal-amplification sites, charge-transport promoters, and anchoring points for recognition elements [27,53]. Noble-metal systems such as Au, Ag, Pt, and Pd generally rely on their high intrinsic conductivity, favorable capacity for surface electronic regulation, and relatively low overpotentials to reduce redox barriers. Among them, Au is particularly suitable for constructing stable immobilization interfaces for antibodies, aptamers, or imprinted layers. Ag can participate in catalysis but can also function directly as an oxidizable or inhibition-responsive signaling entity. Pt and Pd, in contrast, are often employed in core–shell or alloyed structures to amplify surface catalytic activity [54,55,56,57]. By comparison, non-noble-metal systems such as Cu offer the advantages of lower cost, tunable composition, and stronger interactions with phosphorus- and sulfur-containing functional groups, although they are also more susceptible to surface oxidation, aggregation, and insufficient long-term stability. Recent work has therefore moved away from simply comparing which metal affords the highest sensitivity and instead focuses on the deliberate design of monometallic, bimetallic, alloyed, core–shell, and supported composite interfaces to regulate analyte adsorption strength, intermediate stability, and electron-transfer pathways with greater precision, thereby balancing response intensity, selectivity, and stability [53,56,58,59,60].

Nanostructured gold interfaces most clearly illustrate how morphological reconstruction can alter reaction behavior. By constructing a nanostructured gold layer on the electrode surface, Chang and co-workers increased the density of low-coordination active sites, such that the apparent reaction pathway of organophosphorus pesticides was no longer constrained by the scarcity of active sites and mass-transfer limitations characteristic of flat surfaces [54]. The real advance in this type of system therefore lies in the fact that gold nanostructures themselves can reconfigure interfacial reactions through changes in surface morphology and electronic structure. In a different context, the AuNP-triggered immunoelectrochemical platform developed by Talan and co-workers highlights another function of Au: AuNPs act not only as catalytic centers, but also as dual platforms for antibody immobilization and signal transport. Their high surface energy and facile surface functionalization allow more ordered assembly of the recognition layer, thereby expanding the role of metal nanoparticles from surface active sites to recognition-supporting interfaces [55]. Ag- and Cu-based systems, by contrast, are more representative of reaction-type interfaces. Porto and co-workers coupled AgNPs with carbon nanotubes and combined them with multiple-pulse amperometry for the rapid direct analysis of several organophosphorus pesticides, where the combination of detection and cleaning pulses rendered Ag not only an active center but also a dynamically renewable electroactive interface [56]. Zahran and co-workers showed that some pesticides are not detected through their own direct redox processes, but rather through inhibition of the oxidation of AgNPs [57]. Similarly, the laser-scribed electrode modified with copper nanoparticles reported by Bahamón-Pinzón and co-workers demonstrates that CuNPs can also establish effective phosphorus-affinitive interfacial sites on low-cost and printable platforms, with clear potential for field deployment [58].

**Figure 5:**
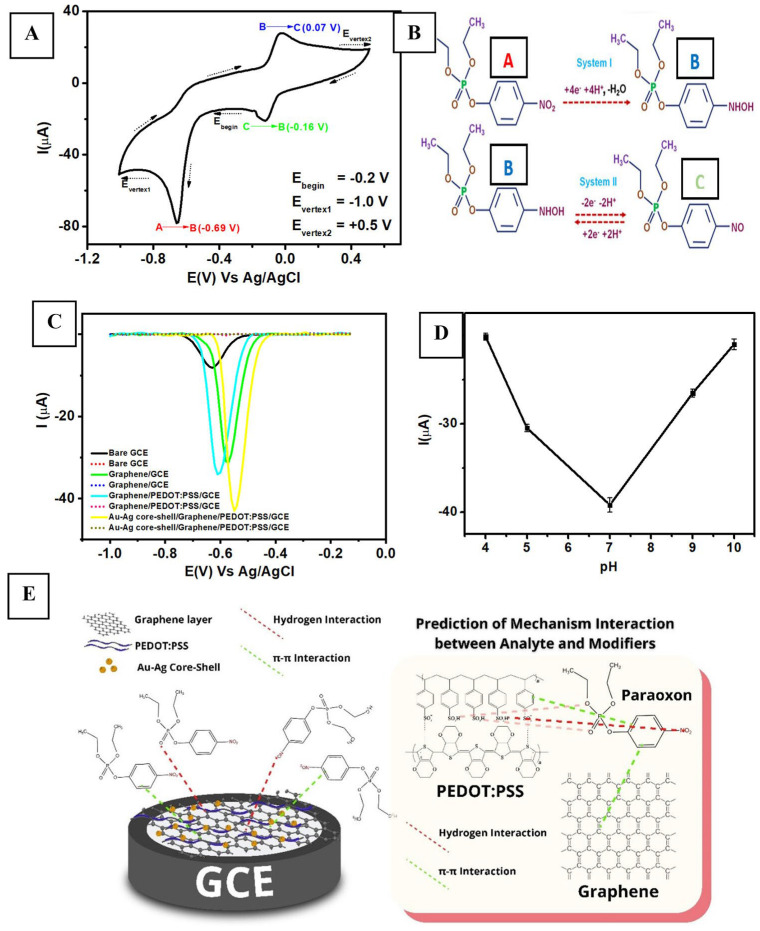
(**A**) Cyclic voltammogram obtained at a scan rate of 50 mV s^−1^ from measuring 1 mM paraoxon in 0.1 M of pH 7 phosphate buffer using bare GCE. (**B**) The complete electrochemical reaction of paraoxon involving its oxidation and reduction processes at bare GCE, (**C**) Voltammograms obtained at a scan rate of 50 mV s^−1^ for the measurements of 100 μM paraoxon-ethyl in 0.1 M of pH 7 phosphate buffer (solid line) and the measurements of electrolyte solution (dashed line). (**D**) The current response (Ipc) of 100 μM paraoxon in 0.1 M of phosphate buffer at a pH range from 4 to 10 measured with Au-Ag core–shell/graphene/PEDOT:PSS/GCE. (**E**) Schematic illustration of the interaction between Au-Ag core–shell, graphene, and PEDOT:PSS on the surface of GCE [59].

The emergence of bimetallic, alloyed, and core–shell systems indicates that the research focus has shifted from “more metallic components” to “better interfacial electronic structures”. Representative electrochemical behavior and the response of the Au-Ag core–shell/graphene/PEDOT:PSS interface are shown in Figure 5. The Au-Ag core–shell/graphene/PEDOT:PSS system developed by Wahyuni and co-workers shows that the significance of the core–shell structure lies not in the simple addition of two noble metals, but in the way the outer Ag shell provides a more reactive surface, the inner Au core stabilizes the interface and tunes the electronic density of the shell, and graphene/PEDOT:PSS establishes a rapid charge-transport network, thereby reconciling surface activity with overall interfacial stability [59]. Earlier Ag/Cu alloy systems had already demonstrated that alloying can alter the adsorption geometry and reduction behavior of Chlorpyrifos (CLPF) at the interface, thereby increasing sensitivity to specific bond-cleavage processes [60].

Although metal nanoparticles have greatly advanced electrochemical sensing for pesticide residues, their practical limits and bottlenecks are equally evident. First, attribution of catalytic activity remains largely empirical. Particle size, morphology, crystal facets, ligands, loading modes, and supports often change simultaneously, making it difficult to rigorously distinguish size effects, facet effects, and alloying effects. Second, although stabilizers and surface ligands help maintain dispersion, they may also impede electron transfer and hinder analyte access, thereby distorting the interpretation of structure-performance relationships. Third, chloride ions, sulfides, organic acids, proteins, and pigments in complex samples can induce surface reconstruction, poisoning, or aggregation of the metallic phase, such that the high sensitivity achieved under laboratory conditions is often difficult to translate reliably into real food and environmental matrices. Bimetallic and core–shell systems additionally face problems of compositional drift, shell inhomogeneity, and surface rearrangement during prolonged use, all of which directly affect batch-to-batch consistency and storage stability [5,27,53,57,59,60]. Future efforts should therefore focus less on the continued pursuit of lower detection limits alone and more on identifying the true active sites through the combined use of in situ characterization and theoretical calculations, while developing morphology-controlled synthetic strategies based on low-ligand or ligand-free conditions.

### 4.4. Metal Compound-Based Materials

Metal compound-based materials enable more programmable regulation of analyte adsorption, activation, and charge transport through valence-state distribution, lattice defects, surface hydroxyl groups, and semiconductor band structures [2,61,62,63,64,65,66]. More importantly, these materials directly influence the thermodynamics and kinetics of interfacial reactions: exposed crystal facets affect adsorption configuration, vacancies alter local electronic density, surface hydroxyls regulate proton-coupled electron transfer, and multivalent centers can act as redox-mediating sites.

**Figure 6:**
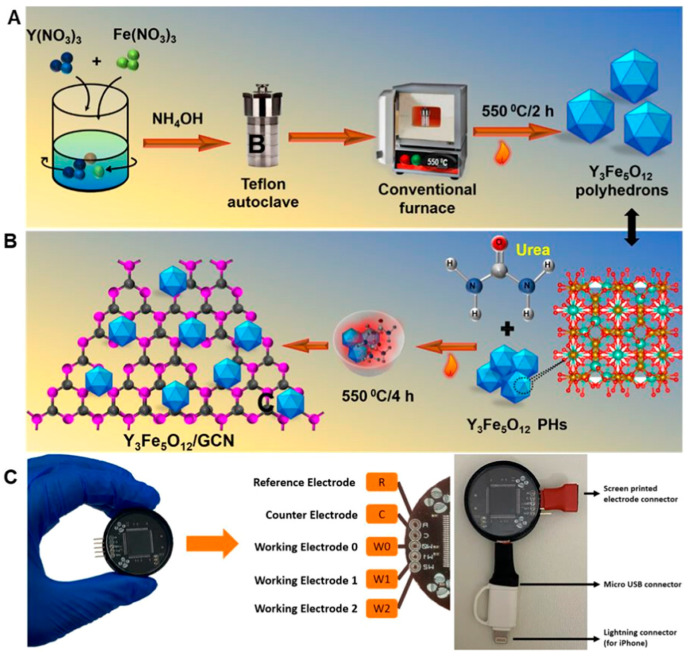
(**A**,**B**) Synthesis of Y_3_Fe_5_O_12_ and Y_3_Fe_5_O_12_/GCN. (**C**) KAUST at miniaturized potentiostat pinouts (**left**) and add-on device format of the KAUST at electrochemical analyzer (**right**) [62].

Wide-bandgap oxides such as TiO_2_ and ZnO rely more strongly on surface hydroxyls, defect sites, and nanoscale effects to promote interfacial enrichment and electron mediation [2,61,62,63,64,65,66]. Transition-metal oxides such as CuO, Co_3_O_4_, NiO, and MnO_2_, by contrast, depend more on reversible multivalent transformations and stronger intrinsic redox activity [2,65,66,67]. Rare-earth molybdates, ferrites, vanadates, and related composite oxides often achieve enhanced electrocatalysis through lattice distortion, ionic-radius mismatch, and oxygen-vacancy induction [62,63,64]. This means that the role of metal oxides in pesticide sensing cannot be reduced to that of a single catalytic active layer; rather, they simultaneously function as semiconducting sensing backbones, defect-regulated catalytic centers, and functional modulatory phases in composite systems. Studies on TiO_2_-based interfaces, Y_3_Fe_5_O_12_/graphitic carbon nitride composites, neodymium molybdate nanostructures, BiOI catalytic strips, Ni-Ce oxide portable interfaces, and Co_3_O_4_:SnO_2_ heterostructures all suggest that sensing performance is often determined less by absolute conductivity than by whether the material can reconfigure the electrochemical reaction coordinates of pesticide molecules through surface chemistry and defect engineering, thereby allowing adsorption, activation, and charge transfer to proceed cooperatively at the same interface [61,62,63,64,65,66,67]. As shown in Figure 6, the Y_3_Fe_5_O_12_/graphitic carbon nitride composite reported by Rajaji et al. provides a representative example, the hybrid interface enabled highly sensitive mesotrione detection in food samples and was further translated into smartphone-enabled point-of-care analysis, underscoring that the value of such oxide composites lies not only in catalytic activity itself, but in the cooperative optimization of interfacial adsorption, charge transfer, and device integration [62].

Metal sulfides and metal phosphides more clearly illustrate the impact of electronic-structure engineering on pesticide sensing. The frequent appearance of MoS_2_ and related sulfides does not simply reflect the continued popularity of two-dimensional materials, but rather the fact that the polarization of M-S bonds, the layered structure, and the abundance of edge sites can simultaneously provide relatively efficient electron transport and stronger surface reactivity. The AgNWs@MoS_2_ system developed by Zheng and co-workers shows that the wire-sheet heterointerface formed between Ag nanowires and MoS_2_ nanosheets can combine low-resistance transport with a high density of edge-active sites, thereby converting the irreversible oxidation of thiabendazole (TBZ) into a more readily readable signal with a higher signal-to-noise ratio [68]. The Pt-doped MoS_2_/Ti_3_C_2_ layered composite platform reported by Wang and co-workers demonstrates that, once coupled with a two-dimensional conductive substrate, sulfides no longer function merely as catalysts, but also simultaneously serve as electron-shunting media, regulators of interfacial polarization, and rapid enrichment platforms [69]. Phosphides represent another route, one more strongly associated with surface polarization and metallic-like conductivity. The MnCoP core–shell structure developed by Karuppusamy and co-workers shows that core–shell configuration can unify the exposure of surface-active sites, redistribution of electronic density, and shortening of diffusion pathways, thereby enhancing the stabilization of reduction intermediates generated during methyl parathion (MP) detection [70].

Layered double hydroxides (LDHs) represent another important branch of metal compound-based sensing interfaces. Their most important advantage lies in interlayer ion-transport channels, the dense distribution of surface hydroxyls, and the possibility of cationic/anionic vacancy engineering through aliovalent doping [71]. NiFe-LDH, NiAl-LDH, and multimetal LDH systems often function as electrocatalytic oxidation phases through in situ transformation into high-valent oxyhydroxides, while their layered architectures help maintain good dispersibility and favorable loading capacity for recognition layers. Recent work on Sn-integrated NiFe-LDH suggests that cation and oxygen-vacancy engineering can extend LDH platforms from single-target response toward more stable simultaneous detection of carbamate pesticides in complex matrices [72].

Metal compound-based materials remain important in electrochemical sensing for pesticides because they integrate adsorption, catalysis, and charge transport within a single inorganic interface. Oxides place greater emphasis on stability, surface hydroxyl chemistry, and multivalent redox cycling; sulfides emphasize rapid electron conduction and highly reactive edge sites; phosphides emphasize surface electronic polarization and the coupling of reactive intermediates; and LDHs display distinctive advantages in interlayer ion migration, vacancy engineering, and parallel detection of multiple targets [2,68,70,71,72]. Nevertheless, several common bottlenecks remain. Discussions of oxygen vacancies, sulfur vacancies, and metal vacancies still largely rest on correlations derived from characterization data rather than on quantitative structure–function relationships. Moreover, it often remains unclear whether the true active sites arise from intrinsic defects, surface reconstruction layers, or oxyhydroxide species generated during operation, as direct in situ evidence is still limited. In real food and environmental samples, these uncertainties are compounded by fouling, matrix-induced surface transformation, and insufficient validation of batch-to-batch consistency and long-term operational stability [5,66,71].

### 4.5. Conducting Polymers

In contrast to carbon materials, which primarily provide continuous conductive frameworks, and inorganic nanophases, which directly supply catalytic active sites, conducting polymers more often function as conductive films, interfacial adhesive layers, supports for molecular imprinting, and microenvironments for the immobilization of biorecognition elements [10,73,74]. Their effectiveness is therefore determined not primarily by nominal electrical conductivity, but by whether the film is continuous, whether the doping state remains stable, and whether the pore structure and surface functionalities permit rapid analyte access and generate electrochemically readable perturbations.

An early study by Du and co-workers, in which a polypyrrole/polyaniline (PPy/PANI) copolymer network was used to encapsulate multi-walled carbon nanotubes (MWCNTs) and immobilize AchE, showed that the primary role of conducting polymers in enzyme sensors is to construct a soft conductive interface that combines electronic coupling with conformational buffering, rather than simply increasing current intensity [75]. Zhang and co-workers used molecularly imprinted PPy on a gold electrode for Gly detection, demonstrating that PPy can simultaneously generate a conductive film and recognition cavities during electropolymerization, thereby enabling even weakly electroactive or non-electroactive pesticides to be indirectly transduced through external probes or changes in interfacial charge transfer [76]. Ding and co-workers advanced this concept by constructing nanotubular imprinted PPy architecture, shifting the strategy from merely having imprinting sites to ensuring that those sites remain accessible and mass transport remains efficient. The thin-walled nanotube structure improved site accessibility, ion migration, and local concentration-gradient-driven transport simultaneously, thereby alleviating the common trade-off between selectivity and rapid response [77]. The study by Balčiūnas and co-workers on a Molecularly imprinted polymer (MIP)-PPy surface-plasmon-coupled interface suggested that competitive adsorption and deposition kinetics among the template molecule, substrate, and electropolymerization process are tightly intertwined. What truly limits the reproducibility of such systems is therefore not simply whether recognition cavities exist, but whether the imprinted layer remains stable, uniform, and regenerable during deposition and template removal [78].

Anirudhan and co-workers constructed a molecularly imprinted copolymer film on MWCNTs using a functionalized thiophene monomer and 3,4-ethylenedioxythiophene (EDOT) for CLPF detection. The real advance in this work did not lie in the higher conductivity introduced by poly(3,4-ethylenedioxythiophene) (PEDOT), but in the fact that the carboxylated thiophene monomer provided specific interactions with the target molecule, EDOT preserved conductivity and film continuity, and MWCNTs overcame the slow binding kinetics and poor template accessibility that commonly affect bulk imprinted polymers [73]. By comparison, the PEDOT/MWCNT system used for mancozeb detection more clearly highlights the function of PEDOT as a continuous electronic network and film-stabilizing phase. Its principal value lies not in recognition, but in integrating otherwise discrete conductive pathways of carbon nanotubes into a coherent film, thereby allowing electrochemical perturbations induced by the target molecule in water samples to be amplified more stably [74].

**Figure 7:**
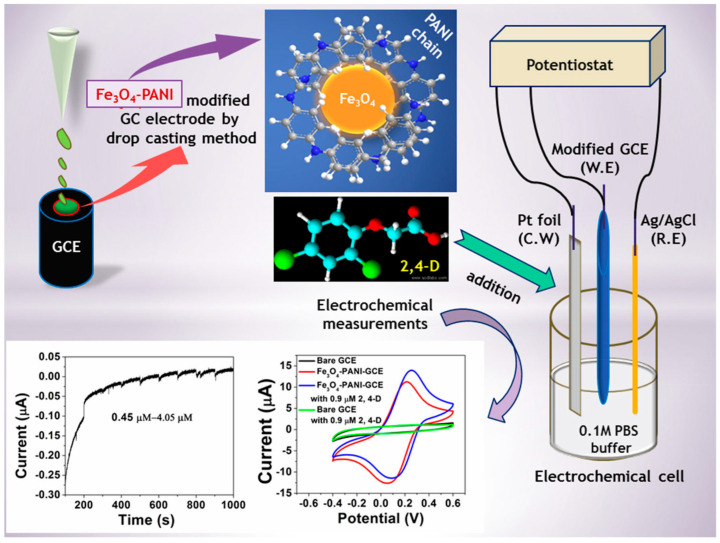
Schematic representation of the glassy carbon electrode (GCE) modification by drop-casting Fe_3_O_4_-PANI and the setup for electrochemical detection of 2,4-Dichlorophenoxyacetic Acid [83].

PANI most clearly exemplifies the dual role of conducting polymers as both electronic coupling bridges and flexible interfacial reinforcement layers. A representative example is the Fe_3_O_4_-polyaniline nanocomposite reported by Goswami and Mahanta (Figure 7), in which PANI served not merely as a conductive additive but as a continuous interfacial matrix that electronically coupled Fe_3_O_4_ nanoparticles and enabled stable non-enzymatic electrochemical detection of 2,4-dichlorophenoxyacetic acid, thereby illustrating how PANI-based composites can simultaneously reinforce charge-transfer continuity and composite-level response stability [79]. In the CuO-PANI interface developed by Sudharsan and co-workers for amperometric CLPF detection, PANI did not merely act as a passive coating; rather, its protonated conjugated network connected otherwise discrete CuO active sites into a continuous reaction interface, while also mitigating the brittleness of the inorganic particles and local accumulation, thereby improving electron transfer and response stability [80]. More recently, the flexible disposable sensing platform based on PANI@S-graphitic carbon nitride (g-C_3_N_4_) further demonstrated that conducting polymers contribute not only to sensitivity, but also directly to device deployment. PANI improved conductive contact among sheet-like materials and enhanced film adhesion, while helping to preserve interfacial continuity on a flexible substrate under bending and in complex sample environments [81]. Similarly, the PA6/PPy nanofibre-rGO system indicates that conducting polymers can, through fibrous and porous structural design, transform films that would otherwise densify readily into three-dimensional interfaces that combine high permeability with mechanical robustness, thereby simultaneously benefiting analyte ingress, electron transport, and film integrity [82].

Overall, the roles of conducting polymers in electrochemical sensing of pesticide residues can be summarized in four aspects: as continuous conductive films that reduce interfacial resistance and stabilize signal output; as flexible adhesive layers that improve the immobilization quality of nanocomponents or recognition molecules; as supports for molecular imprinting that embed selective sites within a conductive backbone; and as electronic coupling bridges in composite systems that coordinate the functional division of labour among inorganic phases, carbon phases, and biorecognition elements [10,73,74,75,76,77,78,79,80,81]. Nevertheless, several important challenges remain. First, the stability of the doping state is still a central bottleneck. In particular, the conductive state of PANI readily drifts under different pH and ionic-strength conditions, whereas PPy films are prone to degradation during overoxidation and template removal [10,74,75,76]. Second, although interfaces rich in functional groups and surface area favour loading of recognition layers, they are also more susceptible to nonspecific adsorption and matrix fouling, especially in fruit juices, fruit and vegetable extracts, and environmental water samples [5,10,77,78,79,80,81]. Third, many high-performance systems are based on multicomponent composites, yet the true origin of the enhanced performance—whether from the doping chemistry of the conducting polymer, the film microstructure, or the catalytic sites of other components—often remains insufficiently distinguished [5,10,79,80,81,82,83]. Future progress will depend less on simply incorporating more components and more on stabilizing polymer doping states, clarifying structure–function relationships under real operating conditions, and integrating conducting-polymer interfaces into reproducible, field-compatible sensing architectures.

### 4.6. MXene-Based Composites

**Figure 8:**
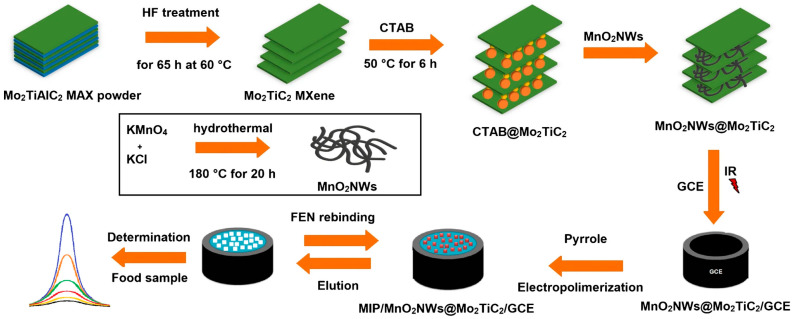
Protocol of MnO_2_ NWs@Mo_2_TiC_2_/GCE nanocomposite preparation and MIP/MnO_2_ NWs@Mo_2_TiC_2_/GCE development [86].

MXenes are most effective in composite systems, where they can coordinate charge transport, surface loading, and interfacial chemistry simultaneously [5,84,85]. Representative MXenes such as Ti_3_C_2_T_x_ possess high electrical conductivity and excellent processability, while their surface terminal groups (-O, -OH, and -F) provide natural anchoring sites for metal nanoparticles, metal compounds, conducting polymers, and biomolecular recognition elements [84,85]. Their real advantage in pesticide detection therefore lies almost entirely in composite architectures rather than in the pristine material itself. When MXenes are used alone, restacking of the sheets compresses the effective surface area, masks accessible active sites, and impairs mass transport. Composite design is therefore not a simple matter of performance addition, but rather a targeted strategy to alleviate intrinsic structural limitations. Yola and co-workers integrating ultrathin MnO_2_ nanowires with Mo_2_TiC_2_ MXene and an electropolymerized molecularly imprinted layer for fenitrothion (FEN) detection are shown in Figure 8, showing that the value of MXene-based composite interfaces lies not merely in accelerating charge transport or preventing sheet restacking, but also in organizing a structurally open and selectively readable microenvironment in which catalytic support, mass transport, and target-specific recognition can be coordinated within the same platform [86].

The Mxene/carbon nanohorns/β-cyclodextrin-MOFs composite interface developed by Tu and co-workers shows that the real role of MXene is not to replace recognition material, but to act as two-dimensional conductive scaffold that integrates host-guest recognition, surface enrichment, and electrochemical signal output within a single low-resistance interface [87]. Particularly representative is the ratiometric sensor developed by Zhong and co-workers based on MXene@Ag nanoclusters and amino-functionalized MWCNTs. In this system, Ag nanoclusters not only suppress MXene-sheet aggregation and improve catalytic activity but are also intentionally designed to provide an internal reference signal. As a result, carbendazim (CBZ) analysis is shifted from a single-peak response to a ratiometric readout, substantially reducing errors arising from fluctuations in film thickness, local loading heterogeneity, and environmental drift [88]. This work shows that the key to MXene-based composite design is not simply to make the surface “more active”, but to use the two-dimensional support to achieve uniform nanocluster anchoring and stable transport, so that signal amplification can be assigned a clear functional role. Similarly, the strategy of self-reduction growth of bimetallic nanoparticles on ultrathin MXene also demonstrates that the surface chemistry of MXene affects not only particle dispersion, but also the organization of the catalytic interface and electron-transfer pathway [89]. The Ti_3_C_2_T_x_-TiO_2_ hierarchical heterostructure likewise indicates that MXene/metal-compound composites do not necessarily rely on direct electrooxidation of the target pesticide. Instead, a heterojunction can be used to regulate the anodic stripping of metal ions, thereby translating pesticide responses that would otherwise be difficult to read with a high signal-to-noise ratio into more stable and amplifiable indirect signals [90]. The Cu_3_V_2_O_8_/Cu_6_Mo_5_O_18_/MXene nanostructure and the Cu_x_O-MWCNTs-COOH/MXene heterostructure confirm that what truly matters is whether interfacial coupling enables simultaneous sheet expansion, active-site exposure, and local redistribution [91,92].

MXene/conducting-polymer and MXene/biological interfaces more directly reveal the value of MXene as an assemblable surface. Once conducting polymers are introduced, their flexible coating, interfacial buffering, and additional π-conjugated pathways often improve interfacial integrity and functionality; biological molecules, in turn, exploit the surface terminal groups of MXene to confer both biofunctionality and assembly capability. The MXene-peptide composite interface reported by Wang and co-workers is particularly illustrative: in this case, the peptide is no longer merely a recognition element, and MXene is no longer merely an inert conductor; instead, the two are coupled through surface chemistry to establish an integrated mode of pre-enrichment, local catalytic degradation, and electrochemical signal response [93]. This result suggests that one of the most important interfacial values of MXene lies in its ability to provide a two-dimensional platform that simultaneously combines electronic coupling, surface tunability, and aqueous-phase stability for the recognition layer, thereby extending pesticide detection from simple electrocatalytic readout toward recognition-enhanced sensing and signal transduction. In conjunction with advances in laser-induced graphene microdroplet electrodes, MXene is also naturally compatible with screen-printed electrodes, laser-induced graphene, and other miniaturized device formats, making it particularly attractive for low-volume samples, rapid on-site testing, and integrated sensing platforms [85,94].

Despite their promise, MXene-based composites still face several important limitations. Although restacking can be alleviated by introducing carbon materials, metal nanoparticles, or oxides as spacers, different composite strategies influence interlayer spacing, terminal-group exposure, and electron transport in different ways, and many so-called “synergistic effects” remain described only at the level of morphology-performance correlation, without more rigorous in situ evidence [85,94]. In addition, the role of MXene surface terminal groups is often oversimplified as merely providing hydrophilicity and facile modification, whereas the relative proportions of -F, -OH, and -O simultaneously influence electronic structure, adsorption energy, and oxidative stability, and their effects are not necessarily uniform across different pesticide molecules [95,96]. Finally, oxidative instability remains a core obstacle to practical application. Many high-performance results are based on freshly prepared dispersions or short-term tests, whereas systematic comparisons of long-term storage stability, batch-to-batch consistency, and surface oxidation/reconstruction in complex matrices are still lacking [95,96].

### 4.7. Others

In addition to carbon-based materials, MOFs and their derivatives, metal nanoparticles, metal compounds, conducting polymers, and MXene-based composites, a series of emerging active materials with clear complementary value has appeared in recent years. Representative directions at this stage mainly include covalent organic frameworks (COFs) and related organic framework materials, conjugated microporous polymers/porous organic polymers (CMPs/POPs), g-C_3_N_4_ and its derived two-dimensional systems, black phosphorus and other layered materials beyond MXenes, as well as supramolecular microenvironment-regulating materials represented by β-cyclodextrin [4,5,97,98,99,100]. The value of these materials lies not primarily in delivering the highest intrinsic conductivity, but in complementing mainstream platforms through pore chemistry, framework conjugation, electronic-structure contrast, or localized microenvironment regulation.

**Figure 9:**
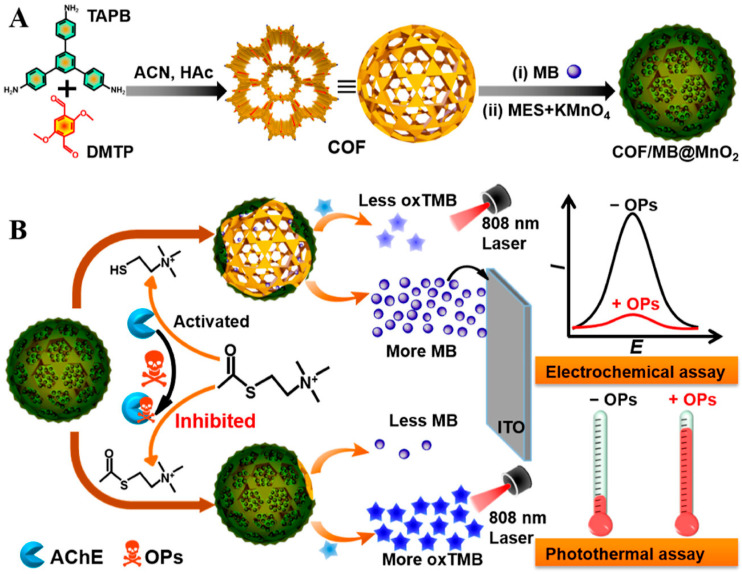
Schematic Illustration of (**A**) the Preparation of COF/MB@MnO_2_ Composite and (**B**) the Mechanism of Complementary Homogeneous Electrochemical and Photothermal Dual-Modal Sensor for OP Detection through Stimuli-Responsive Regulation [97].

The most distinctive feature of COFs and related organic framework materials lies in the programmability of both framework and pore chemistry. Unlike MOFs, which mainly rely on metal nodes to provide open sites, these materials achieve molecular enrichment, sieving, and recognition through covalently constructed periodic organic frameworks, ordered channels, and predefined functional groups [98,99,100,101,102]. Their value therefore arises not primarily from the highest intrinsic conductivity but from the synergy among pore chemistry, framework conjugation, and interfacial functional sites. Particularly representative is the stimuli-responsive COF/methylene blue@MnO_2_ (COF/MB@MnO_2_) composite reported by Wen and co-workers, as shown in Figure 9, in which the porous COF served as a high-capacity carrier for methylene blue while the MnO_2_ shell functioned simultaneously as a thiocholine-responsive gatekeeper and an oxidase-like catalytic layer, thereby coupling signal loading, stimuli-responsive release, and complementary electrochemical/photothermal dual-mode transduction within a single framework platform for reliable organophosphorus pesticide analysis in complex samples [97]. Representative studies on hollow COF/Au nanospheres and free-metal single-COF sensing platforms further indicate that, once pore accessibility and framework functionality are rationally organized, metal loading is no longer the only route to selective and sensitive electrochemical readout [101,102].

The distinctiveness of g-C_3_N_4_ and its derived systems mainly arises from their nitrogen-rich conjugated two-dimensional framework, moderate bandgap structure, and ability to form heterointerfaces with metals or metal oxides [103,104]. Unlike graphene, which is dominated by high-mobility sp^2^ carbon planes, or MXenes, which rely on metallic conductivity and surface terminal groups, g-C_3_N_4_ depends more strongly on the local electronic inhomogeneity, lone-pair electron sites, and tunable surface chemistry associated with triazine or heptazine units, which can be further modulated through doping, vacancy engineering, and compositing. Accordingly, the central role of g-C_3_N_4_ in pesticide sensing is usually not to serve as the strongest conductor, but rather as a highly active two-dimensional interface and an electronic-structure-regulating layer. On the one hand, N sites can enhance the adsorption and coordination of organophosphorus or aromatic pesticides; on the other hand, heterointerfaces constructed with CuO, MXene, and related phases can improve charge separation and interfacial transfer [103,104]. Available studies indicate that the real value of g-C_3_N_4_ does not lie simply in turning a system into a composite but in serving as a source of electronic-structure contrast that transforms a purely adsorptive interface into a cooperative interface integrating adsorption, activation, and transduction.

Black phosphorus and other non-MXene layered materials represent a more frontier direction of exploration. The appeal of black phosphorus lies mainly in its tunable direct bandgap, relatively high carrier mobility, and phosphorus-rich surface bearing lone-pair electrons, which confer molecular adsorption and interfacial charge-response characteristics distinct from those of graphene and MXenes [105,106]. However, black phosphorus has not yet developed into a mature and broadly applicable platform in electrochemical pesticide sensing, primarily because of its poor environmental stability and its susceptibility to oxidative degradation in the presence of oxygen and water, which rapidly deteriorates interfacial performance. As a result, nearly all truly meaningful studies on black phosphorus currently revolve around stabilization and interfacial protection. A representative example is the cofunctionalization of black phosphorene with Au nanoparticles and carboxylated carbon nanotubes for benomyl (BEN) detection, in which carbon nanotubes provide one-dimensional support and electron drainage, Au nanoparticles improve local catalytic activity and surface stability, and black phosphorus contributes a phosphorus-based surface chemistry distinct from conventional carbon interfaces [106].

Beyond framework-based and layered materials, β-cyclodextrin and related microenvironment-regulating layers represent a route that is even closer to interfacial engineering. Their importance lies precisely in the fact that they do not compete based on the highest intrinsic electrocatalytic activity but instead compensate for the shortcomings of conventional materials through host-guest recognition, local wettability regulation, and antifouling interface construction [107,108,109]. The distinctive contribution of β-cyclodextrin is not simply to increase surface area, but to exploit its hydrophobic cavity and hydrophilic outer hydroxyl groups to form selective inclusion interactions, thereby improving the enrichment and recognition of hydrophobic or aromatic pesticides while also enhancing resistance to interference in complex matrices. Relevant studies show that whether in CBZ detection on carbon nanointerfaces or in dinotefuran (DNF) detection on flexible conducting-polymer platforms, the role of β-cyclodextrin extends beyond recognition itself to the construction of a localized microenvironment that integrates flexibility, wettability, and selectivity [108,109,110]. Similarly, conducting microporous organic polymers with functionalized hydroxyl sites and paper-based COF interfaces rich in N and O sites further indicate that the microenvironment layer and the framework layer need not be separated. By integrating host-guest interactions, pore chemistry, and conductive pathways into a single organic framework, more stable recognition and clearer signal readout can be achieved without relying on high metal loading [110,111].

Overall, the common significance of these emerging materials does not lie in immediately establishing a new paradigm capable of replacing mainstream systems, but in compensating for the current limitations of existing material platforms in interfacial selectivity, microenvironmental control, and adaptability to complex matrices. COFs, CMPs, and POPs more tightly integrate the recognition front end with the signal-transduction interface through ordered organic frameworks and pore chemistry. Novel two-dimensional materials such as g-C_3_N_4_ and black phosphorus provide electronic structures and surface chemistries distinct from those of graphene and MXenes. Microenvironmental factors such as β-cyclodextrin further suggest that the key to electrochemical pesticide detection is not always stronger catalysis, but often more rational interfacial selectivity and antifouling design [4,5,97,98,99,100,101,102,103,104,105,106,107,108,109,110,111]. At the same time, these emerging systems also face clear translational limits: many organic framework materials still suffer from insufficient intrinsic conductivity and nontrivial processing reproducibility; black phosphorus remains constrained by oxidation sensitivity and limited environmental robustness; and microenvironment-regulating layers, although conceptually attractive, still require stronger evidence for long-term stability, batch consistency, and matrix-specific antifouling performance under practical operating conditions.

To facilitate a cross-material comparison of the representative sensing platforms discussed in Section 4, the main features of major material categories and specific material subtypes are summarized in Table 2.

## 5. Conclusions and Outlook

Overall, the demands placed on electrochemical sensors for pesticide residue detection have shifted from an early emphasis on rapidity, low cost, and alternative screening toward the development of reliable analytical systems capable of addressing complex samples, stringent regulatory limits, and field deployment. As pesticide classes, application scenarios, and residue forms continue to diversify, the analytical task is no longer confined to trace-level identification of a single target but must simultaneously confront coexisting residues, intensified matrix interference, increasingly strict regulatory thresholds, and the need for portable screening. In this context, the value of electrochemical sensors lies not only in their sensitivity and rapid response but also in their compatibility with miniaturized devices, portable terminals, and intelligent analytical workflows.

As summarized across the material classes and representative examples in Table 2, substantive progress in this field has not arisen from isolated breakthroughs in any single material family. Instead, it has emerged from the gradual evolution of electrode-interface design from empirical stacking toward more deliberate regulation of enrichment, recognition, catalysis, charge transport, and device integration. Carbon materials, MOFs, metal nanoparticles, metal compounds, conducting polymers, MXene-based composites, and selected emerging materials each contribute different strengths, but none of them alone resolves the full set of analytical requirements for practical pesticide monitoring.

At the same time, several contradictions continue to restrict practical translation. Nonspecific adsorption, surface passivation, and matrix interference remain major barriers in real samples; high sensitivity achieved in ideal buffers often does not translate into stable performance in fruit and vegetable extracts, environmental waters, or multiresidue scenarios. Batch-to-batch reproducibility, storage stability, and inter-platform consistency are still insufficiently validated, while scalable fabrication windows and cost-effective field deployment remain underdeveloped for many high-performance interfaces.

Future high-level research should therefore move beyond the simple pursuit of ever-lower detection limits and instead focus on building real analytical systems that are mechanistically clear, sample-robust, and practically manufacturable. Priority directions include standardized fabrication and benchmarking protocols, in situ and operando characterization to identify true active sites and interface evolution, matrix-specific antifouling validation, systematic multiresidue and metabolite analysis, and hybrid portable devices coupled with reliable data processing and intelligent readout.

## Figures and Tables

**Table 1:** Representative pesticide classes relevant to electrochemical sensing.

Pesticides Classification	Examples	General Formulas	Functions
Organophosphorus	Chlorpyrifos (C_9_H_11_C_l3_NO_3_PS) Methyl parathion (C_8_H_10_NO_5_PS) Malathion (C_10_H_19_O_6_PS_2_) Diazinon (C_12_H_21_N_2_O_3_PS) Dimethoate (C_5_H_12_NO_3_PS_2_) Fenitrothion (C_9_H_12_NO_5_PS) Paraoxon-ethyl (C_10_H_14_NO_6_P)	(R_1_O)(R_2_O)P(=X)-Y; X=O or S; Y = leaving group	Mainly insecticides (some also acaricidal/nematicidal); typically acetylcholinesterase (AChE) inhibitors
Carbamates	Carbaryl (C_12_H_11_NO_2_) Carbofuran (C_12_H_15_NO_3_) Methomyl (C_5_H_10_N_2_O_2_S) Methiocarb (C_11_H_15_NO_2_S)	R^1^O-C(=O)-NR^2^R^3^; common N-methyl carbamates: R-O-C(=O)-NHCH_3_	Mainly insecticides; some also nematicidal; typically reversible AChE inhibitors
Pyrethroids	Cypermethrin (C_22_H_19_Cl_2_NO_3_) Deltamethrin (C_22_H_19_Br_2_NO_3_) Permethrin (C_21_H_20_Cl_2_O_3_)	Classical ester pyrethroid motif: R-C(=O)O-CH_2_-Ar; Type II often: Ar-CH(CN)-O-C(=O)-R	Insecticides; fast-acting neurotoxicants, mainly voltage-gated sodium channel modulators
Neonicotinoids	Imidacloprid (C_9_H_10_ClN_5_O_2_) Clothianidin (C_6_H_8_ClN_5_O_2_S) Thiamethoxam (C_8_H_10_ClN_5_O_3_S) Dinotefuran (C_7_H_14_N_4_O_3_)	Heteroaryl + pharmacophore motif, commonly Het-CH_2_-[nitroguanidine/cyanoamidine/nitromethylene]	Mostly systemic insecticides, especially for sucking pests; nicotinic AChR agonists/modulators
Phosphonate herbicides	Glyphosate (C_3_H_8_NO_5_P) Glufosinate (C_5_H_12_NO_4_P)	Representative structure of glyphosate: HOOC-CH_2_-NH-CH_2_-P(=O)(OH)_2_; broader aminophosphonate motif: R-NH-CH_2_-P(=O)(OH)_2_	Broad-spectrum systemic herbicides; glyphosate inhibits EPSPS in the shikimate pathway
Bipyridylium herbicides	Paraquat (C_12_H_14_C_l2_N_2_) Diquat (C_12_H_12_Br_2_N_2_)	Quaternary bipyridinium motif: [(C_5_H_4_N)_2_R]^2+^; paraquat=1,1′-dimethyl-4, 4′-bipyridinium	Non-selective contact herbicides; Photosystem I electron diverters, causing reactive oxygen species formation
Benzimidazole fungicides	Benomyl (C_14_H_18_N_4_O_3_) Carbendazim (C_9_H_9_N_3_O_2_) Thiabendazole (C_10_H_7_N_3_S)	Benzimidazole ring core (fused benzene + imidazole scaffold)	Systemic fungicides; mainly interfere with β-tubulin/tubulin polymerization
Triazole fungicides	Tebuconazole (C_16_H_22_ClN_3_O) Propiconazole (C_15_H_17_Cl_2_N_3_O_2_) Difenoconazole (C_19_H_17_Cl_2_N_3_O_3_) Myclobutanil (C_15_H_17_ClN_4_)	1,2,4-triazole ring-containing scaffold	Systemic fungicides; DMI fungicides targeting C14-demethylase (CYP51)

**Table 2:** Representative electrochemical sensing platforms for pesticide residue detection across major material categories and specific material subtypes.

Material Category	Materials	Analyte (s)	Electrochemical Performance	Refs.
Carbon-based materials	Reduced graphene oxide/1-pyrenebutyric acid/aptamer/screen-printed carbon electrode (SPCE)	Imidacloprid Thiamethoxam Clothianidin	DPV; linear range: 0.01–100 ng·mL^−1^; Limit of detection (LOD): 6.30 pg·mL^−1^ (imidacloprid), 6.80 pg·mL^−1^ (clothianidin), 7.10 pg·mL^−1^ (thiamethoxam); Tomatoes and rice, Recovery rate: 99.0–100.8%; Relative standard deviation (RSD): 1.01–3.39%	[29]
	CNT and Nafion (a conductive polymer)/SPCE	Amitraz	Square wave voltammetric (SWV); Linear range: 0.005–1.00 µg mL^−1^; LOD: 1.0 ng mL^−1^; Honey and longan fruits, Recovery rate: 80.26–103.63%; RSD: 0.62–11.81%	[32]
	Aptamer/Au@HPC (hierarchical porous carbon)/Chit/GCE	Carbofuran (CF)	CV; Linear range: 1.0–100,000 pg/L; LOD: 0.5 pg/L; Celery and rape, Recovery rate: 95.8–99.3%; RSD: 2.6–3.3%	[35]
	hierarchically porous carbon enriched with intrinsic defects/GCE	CBZ	SWV; Linear range: 0.010–1.0 μM; LOD: 0.0061 μM; River water, lettuce, and soil, Recovery rate: 99.3–102.0%; RSD: 1.2–8.3%	[36]
	Mung bean-derived porous carbon@chitosan (CS) composite/GCE	CBZ	DPV; Linear range: 0.1–20 μM; LOD: 20 nM; Apple juice; Recovery rate: 99.4–100.9%; RSD: 2.4–3.4%; Tomato juice, Recovery rate: 98.8–103.2%; RSD: 3.2–4.2%	[37]
	Graphene quantum dots/CS/NiMoO_4_ nanocomposite/GCE	Diazinon (DZN)	DPV; Linear range: 0.1–330 μM; LOD: 27 nM; Cucumber, Recovery rate: 101.3–106.0%; RSD: 1.93–2.16%; Tomato, Recovery rate: 101.0–102.0%; RSD: 2.01–2.85%	[41]
MOFs and derivatives	Hierarchically porous Cu-BTC MOF platform	Gly	DPV; Linear range: 1.0 × 10^−6^–1.0 × 10^−3^ μM and 1.0 × 10^−3^–10 μM; LOD: 1.4 ×10^−7^ μM; Soybean, Recovery rate: 98.0–105.0%; RSD: 2.4–3.7%	[43]
	Amino-modified ionic MOF@CS/AChE	Gly	DPV; Linear range: 1.0 × 10^−9^–1.0 × 10^−3^ μM; LOD: 1.24 × 10^−7^ μM; Strawberries, cucumbers, oilseed rape, and tomatoes, Recovery rate: 87.1–109.6%; RSD: 2.85%	[44]
	Curcumin/UiO-66/GCE	MP	DPV; Linear range: 0.076–76 μM LOD: 0.0037 μM; Tomato, cucumber, peach, Recovery rate: 95.00–111.73%, 95.70–114.80%, 98.69–111.81%; RSD: 3.63–6.91%, 4.40–6.28%, 3.56–8.36%	[45]
	Molecularly imprinted polymer-modified MOF-808/acetylene black/GCE	Dimethoate	DPV; Linear range: 1 × 10^−1^–1 × 10^4^ μM; LOD: 43.05 pM; Tomato, cucumber, peach, Recovery rate: 83.23–100.58%; RSD: 5%	[46]
	Ni-doping nanoporous carbon-graphene composite/GCE	CBZ	DPV; Linear range: 0.04–10.0 µM; LOD: 8.9 nM; Pond water, peach, lemon juices, Recovery rate: 91.3–111%; RSD: 5.7%	[48]
	MOF-derived copper-cobalt oxide decorated on a protonated-gC_3_N_4_ and graphene oxide nanocomposite/FTO	CBZ	SWV; Linear range: 12 mM–10 pM; LOD: 0.63 × 10^−12^ M; Sweet lime and pomegranate, Recovery rate: 98–101%; RSD: 3.8%	[49]
Metal nanoparticles	FTO-AuNPs-anti-chlorpyrifos antibodies	Chlorpyrifos (CLPF)	DPV; Linear range: 1 fM–1 μM; LOD: 10 fM; Apple, pomegranate and cabbage	[55]
	CNT-AgNP/PGE	DZN Malathion (MLT) CLPF	DPV; Linear range: 0.1–20 μM (DZN), 1.0–30 μM (MLT), 0.25–50 μM (CLPF), LOD: 0.354 μM (DZN), 0.894 μM (MLT), 0.533μM (CLPF); Tap water, orange juice, and apple fruit, Recovery rate: 77.0–124.0%	[56]
	Mucilage-AgNP/glassy carbon	CLPF	SWV; Linear range: 0.4–0.62 μM; LOD: 0.049 μM; River water, Recovery rate: 99%	[57]
	Laser-inscribed graphene-Cu	Gly	DC-potential amperometry (DCPA); Linear range: 4–24 µM; LOD: 3.42 ± 1.69 µM; Environmental water, Recovery rate: 98.6–124.8%; RSD: 5.5–37.8%;	[58]
	Au-Ag core–shell/graphene/PEDOT:PSS composite/GCE	Paraoxon-ethyl	DPV; Linear range: 0.2–100 μM; LOD: 10 nM; Chinese cabbage and pear fruit	[59]
	Ag/Cu-graphene/graphene paste electrode	CLPF	DPV; Linear range: 0.01–100 nM; LOD: 400 µM; Well water, Recovery rate: 86.4–95.3%; RSD: 3.8–5.6%; Soil, Recovery rate: 85.6–93.4%; RSD: 3.9–6.2%	[60]
Metal compound-based materials	TiO_2_/CPE	CBZ	SWV; Linear range: 10–420 μM; LOD: 0.017μM; Soil, Recovery rate: 92.5–98.5%; RSD: 2.49–2.69%; Water, Recovery rate: 91–95%; RSD: 2.57–2.75%	[61]
	Yttrium iron garnet/graphitic carbon nitride/SPCE	Mesotrione	DPV; Linear range: 0.005–751.8 μM; LOD: 0.95 nM; Grapes, orange, tomato, guava, and mango, Recovery rate: 95%	[62]
	3D flower-like neodymium molybdate nanosheets/GCE	MP	DPV; Linear range: 0.5–300 μM; LOD: 5.7 nM; Tomato and paddy grain, Recovery rate: 98%; RSD: 2.1%	[63]
	2D BiOI nanostructures/SPCE	Trichlorophenol	DPV; Linear range: 0.01–116 μM; LOD: 0.0019 μM; River Water, Recovery rate: 97.83–98.47%; RSD: 2.40–2.61%	[64]
	NiCeO/GCE	FEN	DPV; Linear range: 1–5747.3 μM; LOD: 1.8 nM; Recovery rates in pond water (96–99%), lake water (92–98%), brinjal (95–99%), and bitter gourd (96–99%); RSD: 3%	[65]
	Ag NWs@MoS_2_/GCE	TBZ	DPV; Linear range: 0.05–10 μM; LOD: 1.75 nM; Pear and apple, Recovery rate: 95.5–103.6%; RSD: 1.98–3.25%	[68]
	Sn-integrated NiFe-LDH/GCE	CF Methiocarb (MTC)	DPV; Linear range: 1–136 μM (CF), 1–171 μM (MTC); LOD: 9.31 nM (CF), 10.71 nM (MTC); Beans, carrots, spinach, cabbage, pond water, and soil, Recovery rate: 96.35–99.96% (CF), 97.01–99.60% (MTC)	[72]
Conducting polymers	surface imprinted conducting polymer@MWCNT/GCE	CLPF	DPV; Linear range: 0.02–1000 nM; LOD: 0.004 nM; Tap water and cucumber, RSD: 1.6–3.2%	[73]
	Molecularly imprinted polypyrrole-modified gold electrode	Gly	DPV; Linear range: 0.03–4.7 nM; LOD: 0.00158 nM; Cucumber and tap water, Recovery rate: 72.7–98.96%; RSD: 1.07–4.48%	[76]
	Molecularly imprinted polypyrrole nanotubes/SPE	Gly	DPV; Linear range: 0.00588–2.06 μM; LOD: 0.114 μM; Recovery rate: 97.45–101.69% (Orange juice), 94.54–102.70% (rice)	[77]
	Fe_3_O_4_-PANI-GCE	2, 4-dichlorophenoxyacetic acid	CV; Linear range: 1.35–2.7 μM; LOD: 0.21 μM; Tomato and paddy grain, Recovery rate: 98%; RSD: 2.1%	[79]
	Cu_x_O-PANI/GCE	CLPF	CV; Linear range: 0.05–12.50 μM; LOD: 9.0 nM; Honey, Recovery rate: 97.84–103%; RSD: 0.24H–2.59%	[80]
	PANI@sulfur-doped graphitic carbon nitride nanosheets/SPE	CBZ	DPV; Linear range: 1–11 pM; LOD: 0.54 pM; Fruit including apple, Chinese pear, guava, and tomato, Recovery rate: 84.6–108.6%; RSD: 0.2–4.1%	[81]
MXene-based composites	MIP/MnO_2_ nanowires/Mo_2_TiC_2_ MXene ionic nanocomposite	FEN	EIS and CV; Linear range: 1.0 × 10^−3^–2.0 × 10^−2^ μM; LOD: 3.0 × 10^−4^ μM; LOQ: 1.0 × 10^−3^μM	[86]
	Mxene/carbon nanohorns/β-cyclodextrin-Metal–organic frameworks/GCE	CBZ	DPV; Linear range: 3.0 nM–10.0 μM; LOD: 1.0 nM; Tomato, Recovery rate: 97.77–102.01%; RSD: 2.7–4.1%	[87]
	MXene@AgNCs/NH_2_-MWCNTs	CBZ	DPV; Linear range: 0.3 × 10^−3^–10.0 μM; LOD: 0.1 nM; Lettuces, Recovery rate: 96.8–100.7%; RSD: 3.6%	[88]
	Ti_3_C_2_T_x_-TiO_2_/GCE	TBZ	DPV; Linear range: 0.3–100.0 nM; LOD: 0.1 nM; Strawberry and paddy water, Recovery rate: 96.4–102.1%; RSD: 4.1%	[90]
	AChE-CS/Cu_3_V_2_O_8_/Cu_6_Mo_5_O_18_/MXene/GCE	MP MLT FEN	DPV; Linear range: 7.6 × 10^−6^–7.6 × 10^−1^ nM, LOD: 3.2 × 10^−7^ nM (MP); Linear range: 3.1 × 10^−5^–3.1 × 10^−3^ nM, LOD: 3.0 × 10^−6^ nM (MLT); Linear range: 3.6 × 10^−4^–36 nM, LOD: 2.3 × 10^−5^ nM; tap water, Recovery rate: 96.6–103.5%; RSD: 0.83–3.61%	[91]
	Hierarchical nano-Cu_x_O decorated MWCNTs-COOH/MXene/GCE	BEN	DPV; Linear range: 10.0 nM to 10.0 μM; LOD: 3.0 nM; apple, Recovery rate: 99.7% to 102.2%; RSD: 3.4%	[92]
	Ti_3_C_2_T_x_/LIG	BEN	DPV; Linear range: 10–6000 nM; LOD: 5.8 nM; Apple, pear, mushroom and environmental water in the fruit and vegetable garden, Recovery rate: 91.6–108.0%	[94]
	MXene-rGO/antibodies against Ed	Endosulfan	DPV; Linear range: 0.0001–1 μM; LOD: 0.497 nM; Leaf extract, root extract and spiked water, Recovery rate: 78.00–94.47%; RSD: 0.50–7.02%	[95]
Others	COF/methylene blue@MnO_2_	CLPF	DPV; Linear range: 0.0014–0.57 nM; LOD: 0.00018 nM; River water and vegetables and apples, Recovery rate: 96.92–104.6%; RSD: 1.58–3.12%	[97]
	AChE/hollow COF_TFPB-DBD_-AuNPs/GCE	Carbaryl	CV; Linear range: 1.10 μM–40.0 μM; LOD: 0.38 μM; Orange juice, Recovery rate: 99.9–101.3%; RSD: 1.6–2.2%	[101]
	TT-COF/GCE	CBZ	DPV; Linear range: 0.005–5 μM; LOD: 2.21 nM; Apple, tomato and pear juice, Recovery rate: 101.8–108.8%; RSD: 0.538–3.359%	[102]
	B-CuO/g-C_3_N_4_/GCE	MLT	DPV; Linear range: 0.000055–0.017 nM; LOD: 0.00363 nM; Apple, tomato, Recovery rate: 87.64–120.59%; RSD: 1.19–2.28%	[103]
	g-C_3_N_4_-Ti_3_C_2_/GCE	MLT	DPV; Linear range: 0.5–90 μM; LOD: 0.07 μM; Potato and tomato, Recovery rate: 96.0–100.2%	[104]
	BP-Au/MWCNTs-COOH/GCE	MLT	DPV; Linear range: 0.007–1.000 µM; LOD: 1.54 × 10^−3^ µM; Apples and grapes, Recovery rate: 97.36–114.3%; RSD: 0.5–5.3%	[106]
	β-cyclodextrin/carbon nanosheets@carbon nanotubes/GCE	CBZ	DPV; Linear range: 0.03–30 μM; LOD: 9.4 nM; Apple, apple juice, Recovery rate: 97.1–99.4%; RSD: 5%	[107]
	Polycaprolactone/polypyrrole/β-cyclodextrin	DNF	DPV; Linear range: 0.2–50 μM; LOD: 0.05 μM; rice, Recovery rate: 96.67–103.65%; RSD: 0.56–1.63%	[108]
	MIP/β-cyclodextrin/activated mung bean-derived carbon/GCE	DNF	DPV; Linear range: 0.05–10 μM; LOD: 0.016 μM; Tea leaves, pear skin, lettuce leaves and insecticidal spray, Recovery rate: 92.0–102%; RSD: 3.2–4.8%	[109]

## Data Availability

No new data was created.
